# Voice and Communication Training Program Improves Performance of University Students in Oral Presentations

**DOI:** 10.1590/2317-1782/20232022146en

**Published:** 2023-12-04

**Authors:** Deborah Cristine Bonetti Rosa, Leonardo Wanderley Lopes, Simone Aparecida Lopes-Herrera

**Affiliations:** 1 Faculdade de Odontologia de Bauru – FOB, Universidade de São Paulo – USP - Bauru (SP), Brasil.; 2 Universidade Federal da Paraíba – UFPB - João Pessoa (PB), Brasil.

**Keywords:** Communication, Training, Voice, Higher Education, Social Skills

## Abstract

**Purpose:**

To investigate the effect of a voice and communication training program for oral presentations on higher education students.

**Methods:**

The proposed training program was based on the areas of social skills, voice projection techniques, and neurolinguistic programming. Thirty-eight students participated in the training with active learning methodologies at the university. Before and after the intervention, the participants recorded a short oral presentation on a topic of their choice. The recording was presented to the other participants and to a panel formed by three examiners (two articulation therapists and a psychologist), who evaluated the oral presentation performances. Moreover, each individual self-assessed their communication. The evaluation criteria covered the linguistic aspects, formal and non-formal, verbal and non-verbal communication, planning, and elaboration of the presentation.

**Results:**

All participants improved their performance in oral presentations regarding verbal and para-verbal aspects, ability to keep the audience, emotional control, planning, objective, content, approach, organization, visual resource, form of presentation, language, and general elements (general presentation).

**Conclusion:**

The proposed training program is effective in improving the performance of university students in oral presentations.

## INTRODUCTION

Public speaking abilities might be a determinant for professional success since it is demanded by the market, becoming a professional skill^(1-4)^. In this context, communication is a fundamental skill to be developed and improved throughout academic and professional trajectory^(5)^. Up to 89.3% of students would prefer that their undergraduate courses offered lessons on how to improve public speaking^(1)^. Most people face difficulties in oral presentations, especially in the academic environment^(5,6)^. The lack of mastering such a skill when presenting seminars leads students to not know how to speak, stand, gesture, or look at the audience. In other words, they do not know how to cope with a situation of exposure^(5-8)^.

A survey involving 2,001 American universities reported an incompatibility between the perception of the newly graduated of their communication skills and the evaluation of their employers. While 80% of the newly graduated considered themselves prepared in terms of communication skills for the work market, only 44% of the employers considered that those skills were adequate for the work market. In addition, such skills predicted a 79.1% chance of a new graduate being hired^(9)^.

Communication takes place in a multimodal way, using verbal, vocal, and non-verbal resources^(9)^. Vocal resources are linked to vocal quality and dynamics, including parameters such as pitch, loudness, intonation, accentuation, modulation, pauses, and rhythm, among others. Verbal resources are speech, the use of words, and the elaboration of speech. Non-verbal resources are body language, complemented by vocal resources and supporting visual signs^(10,11)^.

Communication situations such as public speaking, talking about matters outside our scope, or personal emotional topics might lead anxiety to manifest. Anxiety may emerge in situations of change, new experiences, and other common situations of human development. However, when anxiety is too intense, it can damage professional, academic, and social experiences. Public exposure situations may cause performance anxiety, described as a state of anxiety that emerges in particular conditions and can be considered a reaction to a stimulus. It is a transitory condition that occurs upon the confrontation with a given stimulus. Public speaking, acting, singing, playing a musical instrument in public, or competing in events are known examples of stimuli^(10)^.

In this sense, enhancing the experience and skill of coping with public speaking situations might soften negative impacts^(1,3,8,12)^. Thus, training that allows these experiences and skills might improve performance in presentations, hence reducing complaints.

Neuro-linguistic programming (NLP) is based on neurosciences to emphasize that human behavior originates from neurological processes. It is an important set of skills based on the psychological features of human beings through which individuals achieve the competence of using their skills as much as possible^(13)^. NLP proposes the possibility of programming actions by using the language concerned primarily with reaching results. NLP works by identifying individual patterns, changing their responses to stimuli, and self-regulation. In addition, NLP covers techniques that enable oral presentations by making them common activities that can be performed by students^(14)^.

Based on this, this study aimed to investigate the effect of a voice and communication program on oral presentations in higher education students.

## METHODS

### Study design and ethical questions

The research project of this intervention study was subjected to and approved by the Human Research Ethics Committee (CEP – abbreviation in Portuguese) of the School of Dentistry of – University of São Paulo – FOB/USP, protocol number 2,820,877. All participants signed the informed consent form (TCLE), according to the CEP rules.

### Participants and Location

The convenience sample included 38 higher education students from a public university in the state of São Paulo, 27 females and 11 males aged on average 21.8 years old. All students were in the first year of the Speech-Language Therapy (n=20), Medicine (n=15), and Dentistry (n=3) undergraduate courses.

### Recruitment and application of the initial registration form

To recruit participants, the survey was shared at the university itself (bulletin boards, institutional emails, and website), as well as on social media profiles to which students had access. An e-mail address and telephone number were made available to those interested in taking part in the research. Those who got in touch received a registration form consisting of the following fields: personal details, interest, and willingness to take part in the research along with the necessary information about the research and a report on their main difficulties in oral presentations (such as seminars), as shown in Annex 1.

The participants were selected based on the following inclusion criteria: to be actively enrolled in the first year of undergraduate courses at the institution of origin in the year of the research project; to report difficulties in using linguistic and non-linguistic resources in oral presentations (difficulty in public speaking due to nervousness, in behaving and/or holding the audience’s attention in oral presentations, difficulties with speech intonation, articulation and/or vocal projection only during oral presentations); to be available and accept participating in all the proposed activities. The exclusion criterion was having taken any courses in oral expression or Neuro-linguistic Programming – NLP^(14)^. Before data collection, the participants received a free and informed consent form containing the research calendar (pre-intervention, intervention, and post-intervention) and instructions on the importance of attending all meetings, since otherwise would compromise the results.

### Data collection

#### Assessment

##### Assessment by expert judges

The assessment was carried out through an assessment form named “Oral Presentation Assessment Form – OPF” (Annex 2) based on an oral presentation using PowerPoint on a topic of choice of each participant, which was the same for both the pre- and post-presentation moments. The participant choosing the topic is for us to disregard the technical knowledge and mastery variable and analyze the quality of the oral presentation. Each participant had three minutes for the presentation.

The examiners were calibrated together the week before at a three-hour training meeting, during which the researcher presented the research proposal, all the items on the assessment form were discussed and any doubts were clarified.

The assessment was conducted in two steps: one week before training and one week after the training, considered as pre- and post-intervention, respectively, using the OPF (Annex 2), developed by the researchers and introduced in the item Instruments.

For a blind assessment, three independent examiners were invited. The assessment panel consisted of two articulation therapists, a language specialist (with extensive experience in linguistics), a voice specialist, and a psychologist with experience in social skills.

##### Self-assessment

The oral presentations in the pre- and post-intervention steps were recorded for the self-assessment. The footage was provided to the study participants for them to watch and assess their performance. Along with the footage, we provided the self-assessment form, composed of two parts that should be filled in distinct moments. The footage of the pre-intervention moment was provided one week after the first meeting, and the participants had two weeks to fill out the self-assessment form. The footage of the post-intervention was only provided one week after the last meeting, and the participants had two weeks to fill out the self-assessment form.

The same assessment criterion by the examiners was considered for ruling “improvement, worsening, or neutrality”. The footage was performed in full HD using the Handycam Sony Hdr-CX405 HD equipment and provided to the participants via share drive. Each participant had access only to their respective footage.

### Instruments

#### Assessment form development

The “Oral Presentation Assessment Form – OPF” (Annex 2) was elaborated by the researchers in this study before the start of the intervention, based on protocols of similar assessments^(15)^. Upon literature review, the form needed to be elaborated since no other validated protocols were found that assessed each participant’s performance according to the demands of our study. The presentation must be analyzed by focusing on the items addressed in the support material, which was provided to and worked with the research participants.

The same form was used both in the pre- and post-intervention assessments, and the examiners could not access the pre-intervention form any longer at the post-intervention step to prevent one assessment from influencing the other. The form consists of two parts, both containing qualitative and quantitative criteria since both were assigned numerical values in the final (statistical) analysis, as follows:

**Part I** – The following elements were assessed^(1)^: linguistic criteria (formal and informal aspects) and^(2)^ communication (non-verbal) complementary elements.

The following components of the linguistic criteria were considered: vocabulary, grammar, pronunciation, intonation, pauses, and mean presentation time. The following communication complementary (non-verbal) were considered: articulation*,* loudness*,* eye contact, smiling when suitable, adequate posture, keeping the audience, and emotional control. All these components were subjected to a Likert scale ranging “always” (assigned with a score of 5), “often” (score 4), “sometimes” (score 3), “rarely” (score 2), and “never” (score 1).

**Part II** – Covering the general aspects of oral presentation, to which scores from 0 (zero) to 10 (ten) were assigned, as follows: planning, content, approach, organization, visual resource, Form of presentation, emotional control, language, and general assessment. The analysis criterion was assigned a score between 0 (unprepared) to 10 (excellent).

For an ‘improvement’ to be ruled, the individual must increase at least one level in the Likert scale of the OPF and at least one point in the score by comparing the pre- and post-intervention steps with the General Assessment. In turn, for a ‘worsening’ to be ruled, the individual must decrease a level in the Likert scale and one point in the score. Finally, for ‘neutrality’ to be ruled, the individual must keep the same score based on the examiner’s mean.

#### Self-assessment form development

The first part consisted of the Scale for Self-Evaluation during Public Speaking (Annex 3), an adaptation of the “Self-statements during Public Speaking Scale – SSPS”^(16)^, which aims to self-assess cognitive aspects in situations where public speaking is a stressor. The SSPS is a self-administered instrument consisting of two sub-scales, positive self-assessment, and negative self-assessment, each with five items scored on a scale from zero to five, according to the original description of the scale^(16)^. Participants were asked to fill in the scale as soon as they had finished presenting, as they needed to describe their real feelings about the situation of being exposed in public.

The SSPS scale has two subdivisions: positive (SSPS-P) and negative (SSPS-N) self-affirmations. The participants must choose the score with which they most related, from 0 (strongly disagree with the statement) to 5 (strongly agree with the statement). The items of the SSPS linked to aspects regarded as positive in the self-assessment are those numbered 1, 3, 5, 6, and 9, whereas those regarded as negative are items 2, 4, 7, 8, and 10. Thus, there are ten items on the self-assessment scale (five positive and five negative).

The second part of the self-assessment instrument for this survey was designed by the researchers, based on the second part of the “OPF”, so that the participants and judges could assess the same variables. Participants had to answer this part by assessing their performance after watching their footage. The footage was made available to each participant, and each only had contact with their footage, which was not made available to the others.

#### Intervention

##### Elaboration step

**Figure 1:**
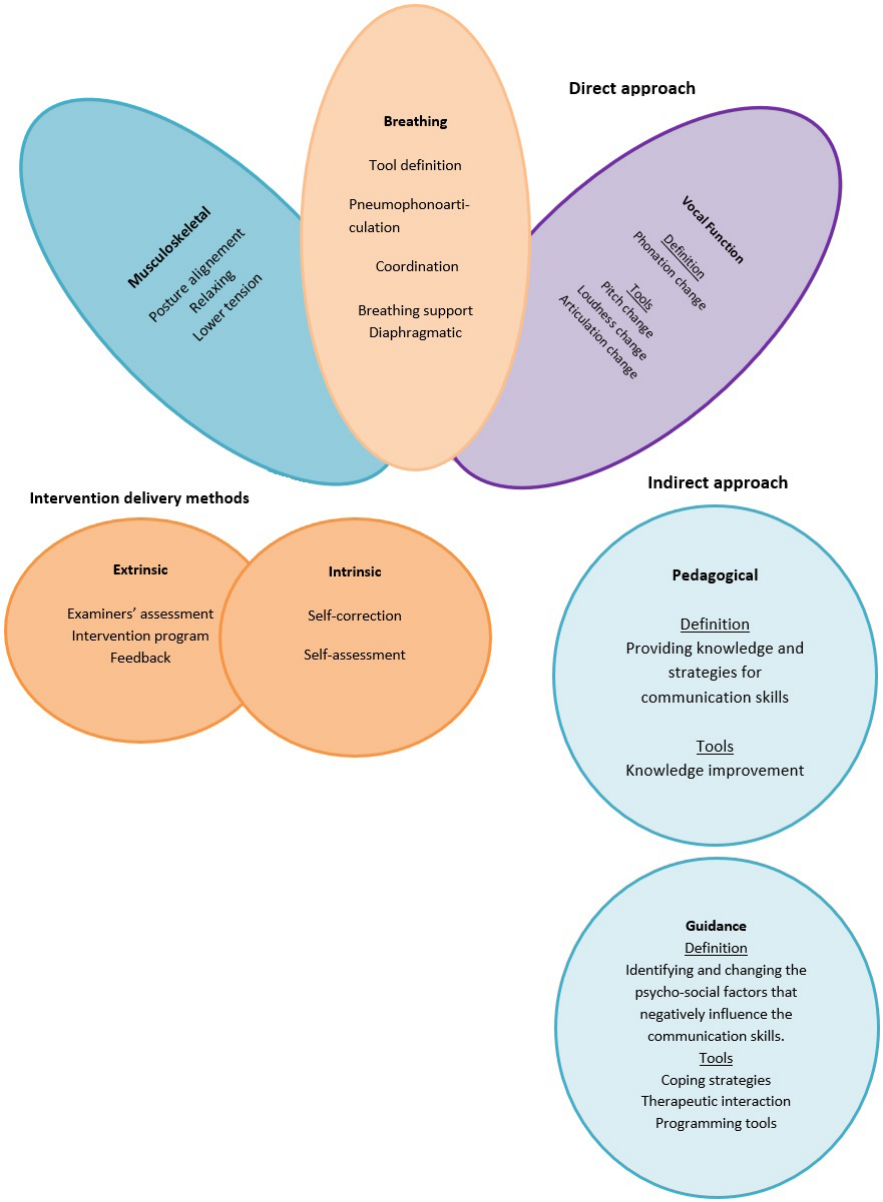
Intervention program taxonomy

We created support material based on a bibliographic survey of effective communication using verbal, vocal, and non-verbal resources, based on social skills training^(13,17,18)^, neurolinguistic programming tools^(14,19)^, and vocal projection and expressiveness techniques^(8,15,17,20)^ as theoretical references. The taxonomic model of communication training used was based (Figure 1) on the vocal therapy taxonomy model by Van Stan et al.^(21)^.

Initially, we carried out a prior pilot study including a smaller number of participants, different from the current sample^(22)^ to test the model for offering the workshop and adapting the support material, as part of a funded scientific initiation project.

The material developed by the authors was freely available to the participants in printed form and as an eBook (in PDF), so that they could access it whenever necessary throughout the training period.

##### Intervention program implementation

This research was conducted over eight weeks at the hours that were convenient to all participants.

The intervention program consisted of ten meetings with theoretical and practical content, weekly, with each meeting lasting an average of two hours. However, the first meeting was held a month before the intervention started to explain the research and sign the consent forms. The last meeting was held to give the participants feedback on their performance. The total number of meetings was to ensure that the participants completed the training within the agreed timeframe of one semester in higher education, as well as based on the results of the abovementioned pilot study and other communication surveys^(1-3)^

The research location was one of the classrooms of the studied university, which is a large space that holds up to 50 students, with mobile chairs for various uses depending on the training dynamics. Chart 1 details the intervention program.

**Chart 1:** Description of the activities and topics covered in the “High-Performance Communication Training”

**Session**	**Objective**	**Content**	**Techniques and Procedures**
1 (Extra)	Explaining the research and sign the terms.	Informed Consent Form (ICF), Term of Commitment, and details of the research.	Performed one month before the intervention for the participants to prepare.
2	Performing the initial assessment (pre-intervention).	First oral presentation on the theme chosen by each participant to the examiners and other participants.	**Assessment.** Application of the “Oral Presentation Assessment Form – OPF”, self-assessment form, and Public Speaking Self-Assessment Scale.
			Each participant had five minutes to conclude the presentation.
3	Developing skills related to the preparation of a presentation. Covering the history of Neurolinguistic Programming, thus discussing some of the theoretical references on which the training is based.	How to search the theme for presentation properly. How to prepare the presentation. How to use audiovisual resources in a presentation and the importance of mastering the content. The history of NLP.	**Indirect approach:** pedagogical intervention.
			**Neuro-linguistic programming.**
4	Teaching how to set goals better and reach results. Explaining the NLP proposal for communication.	NLP tools: good goal formulation – GGF and NLP assumptions.	**Indirect approach**: pedagogical intervention.
			**Neuro-linguistic programming.**
			On this day the participants were asked to write their goals with the training.
5	Briefing on the communication concept and elements of body language, such as facial expressions, gestures, and posture. In addition to social skills and paralinguistic components, which are the vocal components that modulate a message.	Communication, verbal and non-verbal language.	**Direct approach:** body intervention; use of the body to simulate situations; mime.
			**Indirect approach:** pedagogical intervention.
	Providing knowledge on these skills and facing the fear of public speaking.		Therapeutic intervention: Two activities were carried out on this day to work on elements of body language and public speaking highlighting its communicative importance.
6	Increasing knowledge. Addressing the concepts involved in voice projection. Sharing exercises and techniques to improve voice projection in presentations.	Voice projection: body communication, relaxing, breathing, voice, and articulation.	**Indirect approach:** pedagogical intervention. Explaining the theoretical background.
			**Direct approach:** MPT intervention – hearing; vocal, musculoskeletal, somatosensory, and respiratory functions: stretching/relaxation of the cervical region, pectoral girdle, and body; exercises for the semi-occluded vocal tract and articulation. Vocal improvement and expressiveness exercises.
7	Briefing on how to establish a relationship with the public. Explain the types of public according to the NLP and how the content of a presentation should be presented to each one of them. Recognizing the profiles and indicating the prevalence of profiles in the group.	Rapport: how to empathize with the public. Representational systems: how to identify them, what are the features and organizational and learning styles of each group.	**Indirect approach:** pedagogical intervention and therapeutic interaction.
			**Neuro-linguistic programming:**
			Applying the Representational Systems Test.
8	Explaining the relationship between body and mind and providing a larger emotional awareness for better controlling emotions in stressful situations. Showing the importance of choosing words and the role of linguistics.	How to manage emotions and the power of language. It is worth highlighting that the content about emotions was addressed throughout the training. The first sessions approached concepts of emotion, feelings, and self-awareness for the participants to develop their skills throughout the training.	**Indirect approach:** pedagogical intervention and therapeutic interaction.
			**Direct approach:** stretching and relaxation.
			**Neuro-linguistic programming:** dynamics about emotions.
9	Performing the final assessment (post-intervention).	Second oral presentation of the theme chosen by each participant to the examiners and other participants.	**Assessment.** Applying the “Oral Presentation Assessment Form – OPF”, self-assessment form, and Public Speaking Self-Assessment Scale.
			Each participant had five minutes to conclude the presentation.
10 (Extra)	Performance feedback.	Pre- and post-intervention results.	The form contains the individual results for each participant to learn their progress.
		The participants had no contact with the forms filled by the other participants and received their forms via e-mail after the feedback.	

### Data analysis

We performed a descriptive and inferential statistical analysis using the Statistic 10.0 program. Since most of the time the normality test did not show a normal distribution, the Wilcoxon non-parametric test was applied. The significance level of p≤0.05% was adopted.

## RESULTS

Next, we describe the results of 38 participants included for data analysis. We only considered the data from participants who completed the training, with no absences (100% attendance). Although 42 individuals were registered at the start, four (9.5%) of them took the training but had their outcomes disregarded due to two absences throughout the intervention.

### Examiners’ evaluation

The results below describe the assessment by the examiners by comparing the pre- and post-intervention analyses. Table 1 shows the comparison of the qualitative components based on the Likert scale.

**Table 1:** Comparison of the qualitative components according to the moment of evaluation by the evaluators

Qualitative components	Before					After					p-value
	Mean	SD	P25	Median	P75	Mean	SD	P25	Median	P75	
Vocabulary	3.39	0.54	3.00	3.00	4.00	3.92	0.27	4.00	4.00	4.00	<0.001*
Grammar	3.55	0.55	3.00	4.00	4.00	3.92	0.27	4.00	4.00	4.00	<0.001*
Pronoun	3.50	0.60	3.00	4.00	4.00	4.20	0.00	4.00	4.00	4.00	<0.001*
Intonation	3.16	0.59	3.00	3.00	4.00	3.95	0.22	4.00	4.00	4.00	<0.001*
Pauses	2.47	0.68	2.00	2.00	3.00	3.82	0.39	4.00	4.00	4.00	<0.001*
Average time	2.74	0.64	2.00	3.00	3.00	3.87	0.34	4.00	4.00	4.00	<0.001*
Articulation	2.55	0.64	2.00	3.00	3.00	3.79	0.41	4.00	4.00	4.00	<0.001*
Vocal Loudness	3.26	0.79	3.00	3.00	4.00	3.89	0.38	4.00	4.00	4.00	<0.001*
Eye contact	2.26	0.76	2.00	2.00	3.00	3.55	0.55	3.00	4.00	4.00	<0.001*
Smiling when suitable	2.26	0.76	2.00	2.00	3.00	3.79	0.41	4.00	4.00	4.00	<0.001*
Adequate posture	2.21	0.70	2.00	2.00	3.00	3.79	0.41	4.00	4.00	4.00	<0.001*
Keeping the audience	2.53	0.76	2.00	2.00	3.00	3.74	0.44	3.00	4.00	4.00	<0.001*
Emotional control	2.82	0.69	2.00	3.00	3.00	3.79	0.41	4.00	4.00	4.00	**<0.001** *

Wilcoxon Test

* p<0.05

**Caption:** SD=standard deviation; P25=percentile 25; P75=percentile 75

In Table 1, the values of the pre- and post-intervention mean show that the examiners observed a significant improvement in the participants in all items analyzed. The items of eye contact (from 2.26 in the pre-intervention to 3.55 in the post-intervention), smiling when suitable (from 2.26 in the pre-intervention to 3.79 in the post-intervention), and adequate posture during the presentation (from 2.21 in the pre-intervention to 3.79 in the post-intervention) had the greatest impact considering p<0.001 (statistically significant) for all items.

Table 2 shows the comparison of the quantitative components. According to the pre- and post-intervention by the examiners, the following items had the highest improvement means approach (from 7.35 in pre-intervention to 9.28 in post-intervention), presentation organization (from 7.73 in pre-intervention to 9.44 in post-intervention), the language used (from 7.58 in pre-intervention to 9.31 in post-intervention), and Form of presentation (from 7.11 in pre-intervention to 9.07 in post-intervention) – all statistically significant values (p<0.001) for all items.

**Table 2:** Comparison of the quantitative components according to the moment of evaluation by the evaluators

Quantitative components	Before					After					p-value
	Mean	SD	P25	Median	P75	Mean	SD	P25	Median	P75	
Planning	8.11	1.09	7.33	8.42	9.00	9.60	0.59	9.50	9.67	10.00	<0.001*
Objective	7.98	1.02	7.33	8.17	8.75	9.53	0.46	9.33	9.67	9.83	<0.001*
Content	8.00	1.02	7.46	8.08	8.83	9.47	0.56	9.29	9.67	9.83	<0.001*
Approach	7.35	1.07	6.63	7.17	7.88	9.28	0.53	8.96	9.33	9.67	<0.001*
Organization	7.73	1.04	6.96	7.83	8.50	9.44	0.59	9.13	9.54	10.00	**<0.001** *
Visual Resource	8.01	1.04	7.33	8.08	8.88	9.62	0.57	9.50	9.83	10.00	<0.001*
Ways of presenting	7.11	1.03	6.33	7.17	7.67	9.07	0.53	8.73	9.00	9.54	<0.001*
Language	7.58	0.89	7.00	7.67	8.17	9.31	0.41	8.83	9.33	9.67	<0.001*
General	7.71	0.98	7.00	7.83	8.17	9.23	0.48	8.96	9.33	9.58	<0.001*

* p<0.05

Wilcoxon Test

**Caption:** SD=standard deviation; P25=percentile 25; P75=percentile 75

### Self-assessment

The most frequent complaints by the participants regarding public speaking were nervousness (76%), insecurity (74%), anxiety (68%), not being able to convey/express what they want to say (63%), fear of forgetting or making mistakes (60%), followed by shyness (53%), knowing the best posture (53%), being embarrassed to speak in public (50%), speaking clearly (50%), projecting the voice correctly (42%), among others (8%).

The results in Table 3 show a significant increase in the score by comparing the pre- and post-intervention based on the SSPS-P subscale and significantly lower pre- and post-intervention based on the SSPS-N subscale, considering p<0.001 for both. This highlights that the self-assessment not only increased the mean of positive self-affirmations (from 15.9 in the pre-intervention to 20.9 in the post-intervention) but also decreased the mean of negative self-affirmations (from 10.6 in the pre-intervention to 6.3 in the post-intervention). There was also an increase in the percentage of positive self-affirmations, from 44% to 68%, as well as a decrease in negative self-affirmations, from 56% to 32% in the post-intervention.

**Table 3:** Comparison of positive and negative self-affirmations according to the students' moment of self-assessment

Self-affirmations	**Before**			**After**			**P**
	Mean	Median	SD	Mean	Median	SD	
SSPS-P	15.9	16.5	4.9	20.9	22	2.9	**<0.001** *
SSPS-N	10.6	10.5	6.7	6.3	5	4.7	<0.001*

Wilcoxon Test

* *p*<0.05

**Caption:** SD=standard deviation

Table 4 shows the results of the pre- and post-intervention steps in the quantitative self-assessment. Such numbers reveal higher values for the post-intervention compared with the pre-intervention, with statistically significant differences in all items (values of p<0.001). Nonetheless, significantly better means were found for the items of presentation organization (from 6.19 in pre-intervention to 8.06 in post-intervention), the language used (from 6.03 in pre-intervention to 8.11 in post-intervention), most used visual resources (from 6.71 in pre-intervention to 8.53 in post-intervention), emotional control (from 5.17 in pre-intervention to 7.77 in post-intervention), and general assessment (from 6.27 in pre-intervention to 8.30 in post-intervention).

**Table 4:** Comparison of quantitative components according to the moment of student self-assessment.

Quantitative components	Before					After					p-value
	Mean	SD	P25	Median	P75	Mean	SD	P25	Median	P75	
Planning	7.02	1.98	6.00	7.00	8.25	8.53	1.04	8.00	8.00	9.62	**99**
Content	7.35	1.63	6.75	8.00	8.2	8.44	0.80	8.00	8.00	9.00	**<0.001** *
Approach	6.13	2.14	5.00	6.00	8.00	7.66	1.14	7.00	8.00	9.00	<0.001*
Organization	6.19	1.75	5.00	6.00	7.00	8.06	1.16	7.00	8.00	9.00	<0.001*
Visual Resource	6.71	2.24	5.00	7.00	8.00	8.53	1.15	8.00	9.00	9.25	<0.001*
Form of presentation	5.25	2.01	4.00	6.00	7.00	7.60	1.20	7.00	7.00	9.00	<0.001*
Emotional Control	5.17	2.64	3.00	5.00	8.00	7.77	1.41	7.00	8.00	9.00	<0.001*
Language	6.03	1.76	5.00	6.00	7.00	8.11	1.09	7.00	8.00	9.00	<0.001*
General	6.27	1.73	5.75	7.00	7.25	8.30	0.90	8.00	8.00	9.00	<0.001*

* p<0.05

Wilcoxon Test

**Caption**: SD=standard deviation; P25=percentile 25; P75=percentile 75

## DISCUSSION

The proposed training program was effective in improving the communication skills of university students. All variables measured linked to verbal and non-verbal resources improved after the training both by the examiners' and students’ perceptions. It is worth noting that among the qualitative elements analyzed by the examiners, the most highlighted improvement occurred for eye contact, smiling when suitable, and adequate posture during presentation. As to the quantitative aspects, the most highlighted improvement occurred in approach, presentation organization, the language used, and the form of presentation.

Despite the greater improvement perceived by the students in their self-assessments coincided, in qualitative terms, with the examiners’ assessment for the items of presentation organization and language, most of these students perceived a greater improvement in emotional control and their general assessment (including message clarity, achieving goals, interesting dynamics, pleasant/informative or thought-provoking content). Prior research has also reported that the same aspects mentioned by both the examiner and the participants in this study are complementary, which means that usual emotions influence facial expression, voice, and body posture^(9,10)^.

Our study covered techniques and tools from different fields^(13,14,18,19,23-25)^ integrated into a prior didactic material that provided the basis for both the training development and the support material to instruct on the use of linguistic resources in oral presentations for higher education students. Specifically, the items of body posture and facial expression showed a significant improvement, both according to the examiners and the participants. Such a result is based on the mean values reached through greater eye contact, smiling when suitable during the presentation, and body posture by comparing the pre- and post-intervention assessments described herein, which corroborates findings of similar studies in fields related to training and communication^(9,13,14,19)^.

When working with human communication, emotion is an important factor in conveying the intention convincingly, hence being a relevant part of natural, spontaneous human communication. The literature^(11,19,23)^ demonstrates that throughout communication, emotion can be expressed both consciously and unconsciously through elements such as linguistic utterance^(6)^, paralinguistic features, or vocal features^(20)^. Our study corroborates such information more specifically through the participants’ self-assessment, who reported better emotional control by comparing the means before and after the training.

Such a positive self-assessment of the participants’ emotional aspect is significant since their most frequent complaints before starting the intervention, which supported their decision to participate in the training, were nervousness, insecurity, anxiety, and not being able to convey/express what they want to say. According to a prior study^(8)^, these complaints might result from a lack of speaking practice, insufficient knowledge on the topic, and negative self-image, with the lack of experience as the most important factor^(1,23)^, which led the training herein proposed to reach positive outcomes.

Still regarding self-image, the self-assessment by the participants – shown in one of the instruments^(4)^ – indicated that the positive self-affirmations increased significantly. In turn, the negative affirmations about their presentations also decreased significantly, thus reinforcing the results of other studies^(7,8,22)^. Such studies have pointed out that a negative self-image influences oral presentations, whereas when the individual feels prepared, this self-image tends to improve.

Based on such a consideration regarding the self-image of a good communicator, we chose to follow the assumptions of Neuro-linguistic Programming – NLP^(13,14,19)^ as a tool for developing the teaching material and training, in addition to proposing the exercise of mental state change^(18,19,26)^ by directing the effort toward social skills to promote greater confidence and encouraging during the presentation.

To make the change in mental state possible^(19,26)^, the proposed training gave the participants a theoretical introduction to how a presentation should be designed for different types of audiences and the importance of reaching everyone. This concept was later named in the assessment, as in similar studies^(3,12,27)^, as “making eye contact with everyone present” and “ability to maintain the audience’s interest”, considered within the “general” criterion. These aspects were rated similarly in terms of post-intervention improvement by both the judges and the participants themselves, being items with the most significant changes, as shown by the results obtained in studies with similar populations^(3,12,27)^.

The training worked the communication skills through practical activities in which the participants performed voice and body exercises based on the relationship between voice and communication skills pointed out in studies of the field^(17,20,23)^. These activities simulated oral exposition situations of voice projection, pauses, posture, gestures, and other linguistic and communication skills, as suggested in the literature^(3,12,16,20,23,24,27-29)^.

We believe that the improvement in elements such as articulation, loudness, intonation, pauses, and average presentation time reached with the proposed training is linked to the use of specific vocal techniques aimed at, according to studies in the field^(17,20)^, enhancing the articulation pattern, favoring voice projection, softening emission, and^(24,28,29)^ improving the pneumo-phonoarticulatory coordination and promoting greater balance of voice production.

These skills (articulation, loudness, eye contact, smiling when suitable, appropriate posture, keeping the audience, intonation, and pauses) are part of expressiveness^(30)^, and working together with all these variables enables communication that is appropriate to the context and discourse. It also allows them to improve their self-image and the speaker’s communication, which is corroborated in studies not only with similar populations such as university students^(3,12,27)^ but also with other populations, including those involving other professionals^(1,2,7,20)^.

The study participants’ improvement in vocabulary, grammar, and the use of pronouns may have been brought about by the language content covered in the first and last chapters of the theoretical-practical material prepared for the training. The material guided the way of speaking, word choice, and regency so that the students would be aware of these aspects when giving an oral presentation.

For the speaker to have a larger view on their performance, the relationship among all aspects involved in communication must be addressed, such as language, voice, communicative skill, linguistic and non-linguistic genders used when communicating, as well as focusing on their social skills^(2,6,13,17,18,20)^ as observable and measurable behaviors. The focus is moved from merely emotional matters initially reported by the participants, such as fear and anxiety about public speaking^(1,8,11,16,23,30)^.

The self-assessment included in the study brought important results, with several positive implications for the individual, as pointed out in similar studies^(1,3,7,22)^, such as facilitating self-regulation, providing a sense of self-continuity, speeding up the processing of relevant information, helping to set goals, influencing social perception, and contributing to the projection of a consistent and desirable self-image to others^(3)^.

The participants in this study pointed to an improvement in “planning”, “objectives”, “content”, “approach”, “organization”, and “visual resources” in their self-assessment, either because of training addressing how to structure research and presentation – using basic concepts of research methodology and presentation – or because of the focus on the theoretical and practical preparation for presentation. Such a result corroborates other studies reporting that preparing for the topic, like mastering the theme, coherence, scientific background, and Form of presentation, might decrease the fear of public speaking by 75%.

Knowledge of the content directly affects the style the presenter conducts it, demonstrating a greater or lesser degree of confidence and critical handling of the content to engage the audience, as shown in previous works^(25,30)^.

Another aspect to be discussed is the Self-Assessment Scale for Public Speaking (SSPS). Since a limitation of this study was the absence of validated questionnaires that included the items required to assess the participants’ performance according to the demands of the study, the SSPS scale was translated and adapted to the needs of the research.

As for the results of the scale, the scores show that by increasing the SSPS-P (positive self-affirmations) subscale score and decreasing the SSPS-N (negative self-affirmations) subscale score, the participants became more self-confident and able to face the public speaking situation and showed to be less anxious and better prepared after taking the communication training.

Studies^(4,19)^ that used the same scale have highlighted that negative self-affirmations might be more closely associated with anxiety about public speaking than positive self-affirmations. Such a scenario might indicate that by pointing out negative self-affirmations less often, the participants become less prone to reaffirming or increasing their anxiety about public speaking and even evolving into a social phobia or related anxiety disorder. Studies in the field^(8,12-14,19,22,23,26)^ have reported that training for public exposure and communication, such as cognitive-behavioral techniques and social skills, are effective in treating social phobias related to the fear of public speaking.

The results of a study^(27)^ aimed at analyzing a speech therapy intervention for university students in seminar presentations showed positive effects on oral aspects and better confidence perception by the participants when better reaching their audiences. The examiner speech therapists in the above-mentioned study observed advances regarding oral, body, and interaction aspects. Thus, our general results corroborate such findings^(27)^ and open a reflection on why this fear is still one of the most prevalent among university students. Is it not the role of universities to prepare students for this activity?

Every year, thousands of students are admitted to universities in search of a profession, and, during their training, they encounter tasks that demand intellectual growth and the improvement of skills, such as public speaking. If a higher education institution believes that it needs to prepare its students to communicate effectively in their future professional environments^(1-3)^, it is worth reflecting on how it has handled this training.

Should intervention programs on oratory and improving oral communication performance not be part of the professional training curriculum? Training professionals for the job market should focus on teaching skills and abilities beyond the technical content of a given profession. Universities need to provide the conditions for students to learn not only about the content of the profession but also about the behavioral requirements of the job they have chosen^(18)^.

A limitation of this study might be including students from only one educational institution (despite three undergraduate courses). However, as the participants improved their performance in all aspects analyzed after the intervention, the proposed training proved applicable as an auxiliary tool for the planning and improvement of communication training for such a population. Therefore, our research contributes to speech therapists proposing training programs and speech therapy consultancy in the area at universities, representing a promising professional field.

We suggest that similar studies using the training proposed here be developed in other higher education institutions. The goal is that this study can contribute to the teaching of skills and abilities beyond technical content within universities. We also expect this material to be able to integrate subject content at various universities and provide support for university students in the area of communication.

## CONCLUSION

The proposed training program was effective in improving the communication skills of university students in oral presentations. There has been an improvement – from the perspective of both the examiners and the participants – in skills linked to the verbal and non-verbal resources used, as well as in the qualitative aspects, such as eye contact, smiling when suitable, and adequate posture during the presentation. In addition, the quantitative aspects also improved, such as presentation organization, language used, and visual resources.

In the self-assessment, the students participating in the study also reported improved emotional control during the oral presentations and in their overall evaluations after the training, including the clarity of the message, whether the goals were met, and whether it was interesting, enjoyable/informative, or thought-provoking.

## **Annex 1.** First registration form

**Table:**

**Full name:**
**Date of birth: USP Nº:**
**Address:**
**District:**
**City: UF:**
**Telephone: ( ) Cellphone: ( )**
**E-mail:**
**Course: Graduation year:**
**Can you join us for the research in the evening (19-21h), once a week for eight meetings, at xxxxxxxxxxxxxxxxxx, on a day to be arranged?**
**( ) Yes ( ) No**
**On which day of the week would you prefer to take the survey?**
**( ) Monday ( ) Tuesday ( ) Wednesday ( ) Thursday ( ) Friday**
**Please point out your main difficulties in presentations:**
**( ) Nervousness ( ) Anxiety ( ) Insecurity ( ) Shyness ( ) Not being able to convey/express what I want to say ( ) Fear of forgetting or making mistakes ( ) Ashamed of public speaking ( ) Speaking clearly ( ) Projecting my voice correctly ( ) Not knowing the best posture ( ) Other**
**If you point out any others, please describe them here:**

**Who has noticed this difficulty?**
**( ) yourself ( ) a professor ( ) another teacher ( ) classmates ( ) other friends**
**( ) family ( )others/who:**

## **Annex 2**. Examiner’s Evaluation Form - OPF

Oral Presentation

Examiner’s name:

Evaluatee’s name:

Date:

1. Oral presentation evaluation form

Part I – Elements for analysis.

1.1.1. Linguistic criteria – Formal and informal elements:

Adequate use of:

**Table:**

**Frequency**	**Always**	**Often**	**Sometimes**	**Rarely**	**Never**
(a) Vocabulary					
(b) Grammar					
(c) Pronunciation					
(d) Intonation					
(e) Pauses					
(f) Average time					

1.1.2. Communication complementary elements (non-verbal) – use of the elements below:

**Table:**

**Frequency**	**Always**	**Often**	**Sometimes**	**Rarely**	**Never**
(a) Articulation					
(b) Loudness					
(c) Make eye contact with everyone present					
(d) Smiling when suitable					
(e) Adequate posture					
(f) Ability to keep audience interest					
(g) Emotional control					

Part II

From 0 to 10, give a score for each aspect below. 0 being unprepared and 10 being excellent.

**Table:**

**Elements**	**Points for consideration**	**Score**
Planning	Evidence of careful preparation.	
Objectives	Clarity: suitability to the audience or theme.	
Content	Scope, relevance, suitability, knowledge, research, and mastery of the subject.	
Approach	Message, basis, reiteration, variety, and humor.	
Organization	Coherence, clarity, and management.	
Visual resources	Authority, clarity, and management.	
Form of presentation	Rithm, enthusiasm, eye contact, audible articulation, intonation, self-confidence, and body language.	
Language	Clarity, acuity, fluency, appropriateness, pronunciation, and signaling.	
General	Clarity of message. Did it reach the objectives? Is it interesting? Pleasant/informative? Thought-provoking?	

## **Annex 3**. Self-assessment form

Oral Presentation

Name: Date:

Anexo 1. Escala para Auto-Avaliação ao Falar em Público (SSPS) - Versão para o português

**Table:**

Escala para Auto-Avalíação ao Falar em Público Tradução e adaptação para o português: Crippa JAS, Osório F, Graett FG,Zuardi AW, Pinho M, Busano GF, Chaves M, Loureiro SR (2004) Por favor, imagine as coisas que você costuma pensar sobre si mesmo, quando se encontra em alguma situação em que tenha que falar em público. Tendo em mente essas situações, até que ponto você concorda com as afirmações a seguir? Por favor, dê uma nota de O (se você discorda totalmente) a 5 (se você concorda inteiramente com a afirmação)						
1. 0 que tenho a perder? Vale a pena tentar	0	1	2	3	4	5
2. Sou um fracasso	0	1	2	3	4	5
3. Esta é uma situação difícil, mas posso dar conta dela	0	1	2	3	4	5
4. Um fracasso nesta situação seria mais uma prova de minha incompetência	0	1	2	3	4	5
5. Mesmo que não dê certo, não é o fim do mundo	0	1	2	3	4	5
6. Posso dar conta de tudo	0	1	2	3	4	5
7. Qualquer coisa que eu disser vai parecer bobagem	0	1	2	3	4	5
8. Acho que vou me dar mal de qualquer jeito	0	1	2	3	4	5
9. Em vez de me preocupar, poderia me concentrar no que quero dizer	0	1	2	3	4	5
10. Eu me sinto desajeitado e tolo, certamente eles vão notar	0	1	2	3	4	5

*Copyright Stetan G. Hotmann.*

2. General evaluation

From 0 to 10, give a score for each aspect below. 0 being unprepared and 10 being excellent.

**Table:**

Elements	Points for consideration	Score
Planning	Evidence of careful preparation.	
Objectives	Clarity: suitability to the audience or theme.	
Content	Scope, relevance, suitability, knowledge, research, and mastery of the subject.	
Approach	Message, basis, reiteration, variety, and humor.	
Organization	Coherence, clarity, and management.	
Visual resources	Authority, clarity, and management.	
Form of presentation	Rithm, enthusiasm, eye contact, audible articulation, intonation, self-confidence, and body language.	
Language	Clarity, acuity, fluency, appropriateness, pronunciation, and signaling.	
General	Clarity of message. Did it reach the objectives? Is it interesting? Pleasant/informative? Thought-provoking?	
