# Supplementary material for: Voice and Communication Training Program Improves Performance of University Students in Oral Presentations
Source: Codas. 2023 Dec 4;35(6):e20220146. doi: 10.1590/2317-1782/20232022146en (PMC10723579; doi:10.1590/2317-1782/20232022146en)
Supplement: Translation

# Translation: Programa de Treinamento em Voz e Comunicação Melhora o Desempenho de Universitários em Apresentações Orais

**DOI:** 10.1590/2317-1782/20232022146pt

**Authors:** Deborah Cristine Bonetti Rosa, Leonardo Wanderley Lopes, Simone Aparecida Lopes-Herrera

**Affiliations:** 1 Faculdade de Odontologia de Bauru - FOB, Universidade de São Paulo - USP - Bauru (SP), Brasil.; 2 Universidade Federal da Paraíba - UFPB - João Pessoa (PB), Brasil.

**Keywords:** Comunicação, Capacitação, Voz, Educação Superior, Habilidades Sociais

## Abstract

**Objetivo:**

Investigar o efeito de um programa de treinamento em voz e comunicação para apresentações orais em estudantes de ensino superior.

**Método:**

O programa de treinamento proposto foi baseado nas áreas das habilidades sociais, técnicas de projeção vocal e expressividade e a programação neurolinguística, sendo composto de dez sessões com duração média de duas horas cada. Trinta e oito estudantes participaram do treinamento com metodologias ativas de aprendizagem na própria universidade. Antes e após a intervenção, os participantes realizaram uma apresentação oral sobre um tópico da própria escolha, apresentada aos demais participantes e para uma banca formada por três juízes (dois fonoaudiólogos e um psicólogo), que avaliaram o desempenho nas apresentações orais. As apresentações foram gravadas para que cada indivíduo pudesse realizar a autoavaliação da sua comunicação. Os critérios de avaliação foram os aspectos linguísticos formais e não formais, verbais e não verbais, planejamento e elaboração da apresentação.

**Resultados:**

todos os participantes obtiveram melhora de desempenho nas apresentações orais, quanto aos aspectos verbais, não verbais, capacidade de manter a audiência, controle emocional, planejamento, objetivo, conteúdo, abordagem, organização, recurso visual, forma de apresentar, linguagem e geral (apresentação geral).

**Conclusão:**

o programa de treinamento apresentado é efetivo para melhorar o desempenho de universitários durante apresentações orais.

## INTRODUÇÃO

A competência de falar em público pode ser um dos determinantes do sucesso profissional. Faz parte da exigência do mercado de trabalho e passa a ser uma qualificação profissional^(1-4)^. Nesse contexto, a comunicação é uma habilidade essencial a ser desenvolvida e aprimorada durante a vida acadêmica e profissional^(5)^. Demonstra-se que até 89,3% dos estudantes gostariam que seu programa de graduação incluísse aulas para melhorar a modalidade de comunicação em público^(1)^. A maioria das pessoas apresenta dificuldades em se expor em apresentações orais, principalmente no meio acadêmico^(5,6)^. Por não o dominarem é comum que os alunos em situação de seminário não saibam como falar, como se posicionar, gesticular, como olhar para as pessoas, ou seja, não sabem como agir diante da situação de exposição^(5-8)^.

Uma pesquisa com 2.001 universidades americanas demonstrou que havia uma incompatibilidade entre a percepção dos recém-formados quanto às suas habilidades de comunicação e a avaliação dos empregadores. Enquanto 80% dos recém-formados consideravam que estavam preparados em termos de habilidades de comunicação para o mercado de trabalho, apenas 44% dos empregadores consideram que essas habilidades estavam adequadas para o mercado de trabalho. Além disso, observou-se que essas habilidades prediziam em 79,1% a chance de um recém-formado ser contratado^(9)^.

A comunicação acontece de maneira multimodal, por meio de recursos verbais, vocais e não verbais^(9)^. Os recursos vocais são relacionados à qualidade e dinâmica vocal, incluindo parâmetros como *pitch*, *loudness*, entonação, acentuação, modulação, pausas, ritmo, entre outros. Os recursos verbais são a fala propriamente dita, uso de palavras e elaboração do discurso. Os recursos não verbais são a linguagem corporal, sendo complementados pelos recursos vocais e signos visuais de apoio^(10,11)^.

Situações de comunicação como o falar em público, falar de assuntos que não domina ou de conteúdos emocionais relacionados ao lado pessoal são potencialmente propensos à manifestação de ansiedade nas pessoas. A ansiedade pode ocorrer frente a situações de mudanças, de experiências novas, e outras situações habituais do desenvolvimento humano. Porém, quando a ansiedade é muito intensa, ela pode gerar prejuízos na vida profissional, acadêmica e social das pessoas. As situações de exposição em público podem causar a ansiedade de desempenho, descrita como um estado de ansiedade que ocorre em condições particulares e pode ser considerada como uma reação a um estímulo. É uma condição transitória e ocorre durante o confronto com um estímulo específico. Falar em público, atuar, cantar ou tocar um instrumento musical em público ou competir em eventos são exemplos de estímulos conhecidos^(10)^.

Nesse contexto, o aumento da experiência e da capacidade de lidar com situações de falar em público pode suavizar os impactos negativos^(1,3,8,12)^. Portanto, treinamentos que possibilitem essas experiências e o desenvolvimento de tais habilidades podem melhorar o desempenho nas apresentações e, consequentemente, minimizar as queixas.

A programação neurolinguística (PNL) embasada nas Neurociências enfatiza que o comportamento humano se origina de processos neurológicos. A sua importância está no fato de que é um conjunto de habilidades baseadas nas características psicológicas dos seres humanos, por meio das quais os indivíduos obtêm a competência de usar suas capacidades pessoais tanto quanto possível^(13)^. A PNL propõe a possibilidade de programar a ação das pessoas por meio do uso da linguagem, importando-se primariamente com a obtenção de resultados. Atua identificando os padrões das pessoas, alterando suas respostas aos estímulos e sua autorregulação. Além disso, apresenta técnicas que facilitam as apresentações orais, pois torna as atividades habituais e podem ser empregadas pelos estudantes^(14)^.

Com base no exposto, o objetivo do presente estudo foi investigar o efeito de um programa de treinamento em voz e comunicação para apresentações orais em estudantes de Ensino Superior.

## MÉTODO

### Desenho do estudo e questões éticas

Trata-se de um estudo de intervenção, sendo que o projeto da pesquisa foi submetido e aprovado sob número de protocolo 2.820.877 pelo Comitê de Ética em Pesquisa em Seres Humanos (CEP) da Faculdade de Odontologia de Bauru - Universidade de São Paulo - FOB/USP e todos os participantes assinaram o termo de consentimento livre e esclarecido (TCLE), conforme normas do CEP.

### Participantes e local

Foi constituída uma amostra por conveniência de 38 estudantes de Ensino Superior de uma Universidade Pública do Estado de São Paulo, sendo 27 do sexo feminino e 11 do sexo masculino com a faixa etária média de 21,8 anos. Todos alunos do 1º ano de Graduação dos cursos: Fonoaudiologia (n=20), Medicina (n=15) e Odontologia (n=3).

### Recrutamento e aplicação da ficha de cadastro inicial

Para recrutar os participantes, a pesquisa foi divulgada na própria universidade (murais, emails institucionais, site), assim como foram utilizadas divulgações nas redes sociais em perfis aos quais os alunos tivessem acesso. Foi disponibilizado um e-mail e telefone para contato aos interessados em participar da pesquisa. Aos que entraram em contato, foi disponibilizada uma ficha de cadastro composta pelos campos: dados pessoais, interesse e disponibilidade em participar da pesquisa com as informações necessárias sobre sua realização e, por último, o participante tinha que relatar quais suas principais dificuldades nas apresentações orais (como seminários, por exemplo), conforme ficha de cadastro disponível no Anexo 1.

Para selecionar os participantes, foram estabelecidos os seguintes critérios de inclusão: cursar, com matrícula ativa, o primeiro ano dos Cursos de Graduação na instituição de origem no ano de desenvolvimento da pesquisa; relatar dificuldades em usar recursos linguísticos e não linguísticos para se apresentar oralmente (dificuldade em falar em público devido ao nervosismo, em se portar e/ou prender a atenção da plateia em apresentações orais, dificuldades de entonação de fala, articulação e/ou projeção vocal presentes apenas em momentos de apresentação oral); ter disponibilidade e aceitar participar de todas as atividades propostas. O critério de exclusão foi ter realizado algum curso de expressão oral ou Programação Neurolinguística - PNL^(14)^. Foi entregue um termo de compromisso para os participantes com o calendário da pesquisa (pré-intervenção, intervenção e pós-intervenção) antes da coleta de dados iniciar, explicando a importância de não haver falta em nenhum dos encontros, dado o comprometimento que isso acarretaria nos resultados do estudo.

### Coleta de dados

#### Avaliação

##### Avaliação dos juízes especialistas

A avaliação foi realizada por meio de uma ficha de avaliação denominada “Formulário de avaliação da apresentação oral - FAO” (Anexo 2), com base em uma apresentação oral sobre tópico da própria escolha em PowerPoint, previamente selecionada por cada participante, sendo o mesmo para os momentos de avaliação pré e pós-intervenção. Justifica-se a escolha do tema ser realizada pelo participante para se excluir a variável conhecimento e domínio técnico e observar a qualidade da apresentação oral. Cada participante teve três minutos para apresentação.

A calibração dos juízes foi realizada na semana prévia em conjunto, em reunião de treinamento com duração de três horas, na qual a pesquisadora apresentou a proposta da pesquisa e foram discutidos todos os itens do formulário de avaliação e esclarecidas as dúvidas.

A avaliação foi realizada em dois momentos: uma semana antes do início do treinamento e uma semana após o final do treinamento, considerados como pré e pós-intervenção, com uso do FAO (Anexo 2), construído pelas pesquisadoras e explicado no item Instrumentos.

Para que a avaliação fosse de forma cega, foram convidados três juízes não autores do estudo. Dessa forma, a banca avaliadora foi formada por dois fonoaudiólogos, um especialista na área de linguagem (com vasta experiência na área de Linguística), outro especialista em voz e um psicólogo com experiência em habilidades sociais.

##### Autoavaliação

Também foram realizadas filmagens das apresentações orais realizadas nos momentos de pré e pós-intervenção para a autoavaliação. As filmagens foram disponibilizadas aos participantes do estudo para que pudessem assistir e julgar seu desempenho. Junto às filmagens, foi disponibilizado um formulário de autoavaliação, que possuía duas partes, as quais deveriam ser preenchidas em momentos diferentes. As filmagens do momento pré foram disponibilizadas uma semana após o primeiro encontro, e os participantes tiveram o prazo de duas semanas para preencher o formulário de autoavaliação. As filmagens do momento pós foram disponibilizadas uma semana após o último encontro, e os participantes também tiveram o prazo de duas semanas para o preenchimento.

Para considerar “melhora, piora ou neutralidade”, considerou-se o mesmo critério da avaliação dos juízes. As filmagens foram realizadas com o equipamento Handycam Sony Hdr-CX405 HD, em full HD e disponibilizadas aos participantes via drive de compartilhamento. Cada participante tinha acesso apenas às suas próprias filmagens.

### Instrumentos

#### Construção do formulário de Avaliação

O “Formulário de avaliação da apresentação oral - FAO” (Anexo 2) foi elaborado pelas pesquisadoras deste estudo antes do início da intervenção, baseado em protocolos de avaliação semelhantes^(15)^. Foi necessária a elaboração desse formulário, pois não foram encontrados protocolos validados que avaliassem a performance de cada participante de acordo com a necessidade deste estudo no momento da revisão de literatura realizada. Tal fato se justifica uma vez que a análise da apresentação teria que focar os itens trabalhados no material de apoio que foi disponibilizado e trabalhado com os participantes da pesquisa.

O mesmo formulário foi utilizado na avaliação pré e pós-intervenção e os avaliadores não tiveram mais contato com o formulário da pré-intervenção no momento da pós-intervenção, para que não houvesse influência de uma avaliação sobre a outra. O formulário apresenta duas partes, ambas com critérios qualitativos e quantitativos, dado que a ambos foram atribuídos valores numéricos na análise final (estatística), sendo:

**Parte I** - em que constam os seguintes aspectos a serem avaliados^(1)^: Critérios linguísticos (aspectos formais e informais) e^(2)^ Aspectos complementares à comunicação (não-verbais).

Os componentes que compuseram os critérios linguísticos considerados foram Vocabulário, Gramática, Pronúncia, Entonação, Pausas, Média de Tempo da apresentação. Os aspectos complementares à comunicação (não-verbais) considerados foram Articulação*, Loudness,* Contato Visual, Sorrir quando apropriado, Postura Adequada, Manter a Audiência, Controle Emocional. Para esses, o critério de análise era uma Escala Likert que variava de “Sempre” (ao qual de atribuía o valor 5), “Frequentemente” (valor 4), “As vezes” (valor 3), “Raramente” (valor 2) e “Nunca” (valor 1).

**Parte II** - em que constam os aspectos gerais da apresentação oral, aos quais foram atribuídas notas de 0 (zero) a 10 (dez), que foram Planejamento, Conteúdo, Abordagem, Organização, Recurso Visual, Forma de Apresentação, Controle Emocional, Linguagem, Avaliação Geral. O critério de análise era a atribuição de uma nota de 0 (despreparado) a 10 (excelente).

Para ser considerada “melhora” o indivíduo deveria subir pelo menos um nível da escala de Likert na FAO e o mínimo de um ponto na nota, comparando pré e pós-intervenção na Avaliação Geral. Para considerar “piora” no desempenho, deveria diminuir um nível na escala Likert e um ponto na nota e, para ser considerado neutro, deveria se manter na mesma pontuação, considerando a média dos juízes.

#### Construção do formulário de Autoavaliação

A primeira parte foi composta pela Escala para Autoavaliação ao Falar em Público (Anexo 3), adaptação do “Self Statements during Public Speaking Scale - SSPS”^(16)^, que visa à autoavaliação dos aspectos cognitivos em situações que tenham como estressor o falar em público. A SSPS é um instrumento autoaplicável, composto de duas subescalas, a de autoavaliação positiva e a de autoavaliação negativa, cada qual com cinco itens pontuados numa escala de zero a cinco, de acordo com a descrição original da escala^(16)^. Foi solicitado aos participantes que preenchessem a escala assim que terminassem de apresentar, pois era necessário que o estudante colocasse o real sentimento diante daquela situação, que é a de exposição em público.

A escala SSPS possui duas subdivisões, sendo elas autoafirmações positivas (SSPS-P) e negativas (SSPS-N). Os participantes deviam assinalar cada questão com a nota que mais se identificassem, indo de 0 (discordo totalmente com a afirmação) a 5 (concordo inteiramente com a afirmação. Os itens da SSPS vinculados a aspectos considerados positivos na autoavaliação são os numerados 1, 3, 5, 6 e 9; os itens considerados negativos são os itens 2, 4, 7, 8 e 10, sendo, portanto, considerados dez itens na escala de autoavaliação (cinco de caráter positivo e cinco de caráter negativo).

A segunda parte do instrumento de autoavaliação dessa pesquisa foi elaborada pelas pesquisadoras, com base na segunda parte do “FAO”, para que os participantes e juízes pudessem avaliar as mesmas variáveis. Os participantes deveriam responder essa parte julgando seus desempenhos após assistirem suas próprias filmagens. As filmagens eram disponibilizadas a cada participante, sendo que cada um tinha contato apenas com a sua filmagem, não sendo disponibilizadas aos demais.

#### Intervenção

##### Etapa de elaboração

Foi elaborado um material de apoio realizado a partir de um levantamento bibliográfico, sobre comunicação efetiva usando os recursos verbais, vocais e não verbais que utilizou como referencial teórico o treino das habilidades sociais^(13,17,18)^, ferramentas da programação neurolinguística^(14,19)^ e técnicas de projeção vocal e expressividade^(8,15,17,20)^. O modelo taxonômico de treinamento de comunicação utilizado foi baseado (Figura 1) no modelo de taxonomia de terapia vocal de Van Stan et al.^(21)^.

**Figura 1:** Taxonomia do programa de intervenção

Inicialmente, foi realizado um estudo piloto com um número reduzido de participantes diferentes da amostra atual dessa pesquisa, que precedeu esse estudo^(22)^, para que fosse previamente testado o modelo de oferta da oficina e adaptação do material de apoio, como parte de um projeto de iniciação cientifica com fomento.

O material elaborado pelas autoras foi disponibilizado gratuitamente para os participantes em formato impresso e também em formato ebook (em pdf), para que pudessem acessá-lo quando necessário ao longo do período de treinamento.

##### Implementação do programa de intervenção

A pesquisa foi realizada num período de oito semanas, sendo o horário viável a todos os participantes da pesquisa.

O programa de intervenção foi constituído por dez encontros com conteúdos teórico-práticos, em regime semanal, com duração média de duas horas cada encontro. Contudo, o primeiro encontro foi realizado um mês antes de a intervenção iniciar, para a explicação da pesquisa e assinatura dos termos de consentimento. O último encontro foi realizado para o feedback do desempenho aos participantes. O número total de encontros justifica-se para que os participantes concluíssem o treinamento dentro do tempo estipulado de um bimestre no Ensino Superior, e tendo como base também os resultados do estudo piloto mencionado e outros estudos de comunicação realizados^(1-3)^

O local de realização foi uma das salas de aula da universidade, constituída por um espaço amplo, com lugares para até 50 alunos, cadeiras móveis permitindo uso variável, dependendo das dinâmicas realizadas no treinamento. O detalhamento do programa de intervenção está disponível no Quadro 1.

**Quadro 1:** Descrição das atividades e temas abordados no “Treinamento de Alta Performance em Comunicação”

**Encontro**	**Objetivo**	**Conteúdo**	**Técnica e Procedimentos**
1º (Extra)	Explicar sobre a pesquisa e assinatura de termos.	Termo de Consentimento livre esclarecido (TCLE), Termo de compromisso e detalhamento da pesquisa.	Realizado um mês antes do início da intervenção, para que os participantes pudessem se preparar.
2º	Realizar avaliação inicial (pré-intervenção).	Primeira apresentação oral do tema escolhido por cada participante para a banca e os demais participantes.	**Avaliação.** Aplicação do “Formulário de avaliação da apresentação oral - FAO”, ficha de autoavaliação e Escala para Autoavaliação ao Falar em Público.
			Para apresentação cada participante teve 5 minutos para conclusão.
3º	Desenvolver as habilidades relacionadas a elaboração de uma apresentação. Abordar a história da Programação neurolinguística, dissertando assim sobre um dos referenciais teóricos que embasou o treinamento.	Como realizar a busca adequada do tema a ser apresentado; como elaborar a apresentação; como utilizar os recursos audiovisuais em uma apresentação e a importância do domínio do conteúdo. E a História da PNL.	**Abordagem indireta:** intervenção pedagógica.
			**Programação Neurolinguística.**
4º	Ensinar a como definir melhor seus objetivos e alcançar os resultados. E explanar sobre o que a PNL propõe em relação à comunicação.	Ferramentas da PNL: Boa Formulação de Objetivos - BFO e Pressupostos ou Pressuposições da PNL.	**Abordagem indireta**: intervenção pedagógica.
			**Programação Neurolinguística.**
			Nesse dia foi solicitado aos participantes que escrevessem seus objetivos e metas com o treinamento.
5º	Instruir sobre o conceito de comunicação e aspectos sobre linguagem corporal, como expressões faciais, gestos e postura. Além das habilidades sociais e os componentes paralinguísticos, que são os componentes vocais que modulam uma mensagem.	Comunicação, Linguagem verbal e não verbal.	**Abordagem direta:** Intervenção corporal; uso do corpo para simular situações; mimica.
			**Abordagem indireta:** intervenção pedagógica.
	Propiciar o conhecimento dessas habilidades e enfrentamento do medo de se expor em público.		Interação terapêutica: Nesse dia foram realizadas duas atividades que trabalharam aspectos da linguagem corporal e exposição em público, dando ênfase à sua importância comunicativa.
6º	Aumento de conhecimento. Abordar os conceitos envolvidos na projeção vocal.	Projeção Vocal: Comunicação corporal, propriocepção, relaxamento, respiração, voz e articulação.	**Abordagem indireta:** intervenção pedagógica. Realizado a explicação do conteúdo teórico
	Vivenciar os exercícios e técnicas para melhorar a projeção vocal nas apresentações.		**Abordagem direta:** Intervenção MPT - auditiva; função vocal, musculoesquelética, somatossensorial e respiratória: alongamento / relaxamento da região cervical, cintura peitoral e corpo; exercícios de trato vocal semiconcluído e articulação. Exercícios de aprimoramento vocal e expressividade.
7º	Orientar sobre como estabelecer uma relação com o público. Explicar quais tipos de públicos existem de acordo com a PNL e como deve ser apresentado o conteúdo de uma apresentação a cada um deles.	Rapport, como estabelecer empatia com o Público. Os Sistemas Representacionais, como identificar, quais as características, estilos de organização e aprendizagem de cada grupo.	**Abordagem indireta:** intervenção pedagógica e interação terapêutica.
			**Programação Neurolinguística:**
	Reconhecimento dos perfis e indicar a prevalência dos perfis no grupo.		Aplicação do Teste dos Sistemas Representacionais.
8º	Explanar sobre a relação entre corpo e mente propiciar maior consciência emocional para ter melhor controle sobre as emoções diante dessas situações de estresse. Ilustrar sobre a importância da escolha de palavras e o papel da linguística.	Foi abordado o conteúdo sobre como trabalhar as emoções e o Poder da Linguagem. Ressalta-se que o conteúdo sobre emoções foi trabalhado durante todo o treinamento, e nos primeiros encontros foram trabalhados conceitos de emoção, sentimento e autoconhecimento, para que os participantes pudessem desenvolver essas habilidades durante todo o treinamento.	**Abordagem indireta:** intervenção pedagógica e interação terapêutica.
			**Abordagem direta:** alongamento e relaxamento
			**Programação Neurolinguística:** dinâmica sobre as emoções.
9º	Realizar avaliação final (pós-intervenção).	Segunda apresentação oral do tema escolhido por cada participante para a banca e os demais participantes.	**Avaliação.** Aplicação do “Formulário de avaliação da apresentação oral - FAO”, ficha de autoavaliação e Escala para Autoavaliação ao Falar em Público.
			Para apresentação cada participante teve 5 minutos para conclusão.
10º (Extra)	Feedback sobre o desempenho	Resultados pré e pós-intervenção.	Ficha com os resultados individuais para que cada participante pudesse ver sua evolução.
		Os participantes não tiveram contato com as fichas dos demais participantes e receberam as suas respectivas fichas por e-mail após o feedback.	

### Analise de dados

A análise estatística foi realizada de forma descritiva e inferencial. Utilizou-se o Statistic 10.0. Foi aplicado o teste de normalidade e, pela grande maioria das variáveis não ter apresentado distribuição normal, foi utilizado o teste não-paramétrico, Teste de Wilcoxon. O nível de significância adotado para os resultados deste estudo serem considerados significantes foi de p≤0,05%.

## RESULTADOS

A seguir serão descritos os resultados dos 38 participantes considerados na análise de dados. Só foram considerados os dados dos participantes que cumpriram o treinamento na totalidade, sem faltas (100% de presença). Embora no início, houvesse 42 inscritos, 4 (9,5%) deles realizaram o treinamento, mas tiveram os dados desconsiderados por terem uma ou duas faltas durante o período da intervenção.

### Avaliação dos juízes

Os resultados a seguir ilustram a avaliação realizada pelos juízes, comparando a análise pré e pós-intervenção. A Tabela 1 é a comparação dos componentes qualitativos, considerando a escala Likert.

**Tabela 1:** Comparação dos componentes qualitativos em função do momento de avaliação dos avaliadores/juízes

Componentes qualitativos	Pré					Pós					Valor-p
	Media	DP	P25	Mediana	P75	Media	DP	P25	Mediana	P75	
Vocabulário	3,39	0,54	3,00	3,00	4,00	3,92	0,27	4,00	4,00	4,00	<0,001*
Gramática	3,55	0,55	3,00	4,00	4,00	3,92	0,27	4,00	4,00	4,00	<0,001*
Pronome	3,50	0,60	3,00	4,00	4,00	4,20	0,00	4,00	4,00	4,00	<0,001*
Entonação	3,16	0,59	3,00	3,00	4,00	3,95	0,22	4,00	4,00	4,00	<0,001*
Pausas	2,47	0,68	2,00	2,00	3,00	3,82	0,39	4,00	4,00	4,00	<0,001*
Média de Tempo	2,74	0,64	2,00	3,00	3,00	3,87	0,34	4,00	4,00	4,00	**<0,001** *
Articulação	2,55	0,64	2,00	3,00	3,00	3,79	0,41	4,00	4,00	4,00	<0,001*
Loudness Vocal	3,26	0,79	3,00	3,00	4,00	3,89	0,38	4,00	4,00	4,00	<0,001*
Contato Visual	2,26	0,76	2,00	2,00	3,00	3,55	0,55	3,00	4,00	4,00	<0,001*
Sorrir quando apropriado	2,26	0,76	2,00	2,00	3,00	3,79	0,41	4,00	4,00	4,00	<0,001*
Postura Adequada	2,21	0,70	2,00	2,00	3,00	3,79	0,41	4,00	4,00	4,00	<0,001*
Manter a Audiência	2,53	0,76	2,00	2,00	3,00	3,74	0,44	3,00	4,00	4,00	<0,001*
Controle Emocional	2,82	0,69	2,00	3,00	3,00	3,79	0,41	4,00	4,00	4,00	<0,001*

Teste de Wilcoxon

* p<0,05

**Legenda**: DP=desvio padrão; P25=percentil 25; P75=percentil 75

Na Tabela 1 fica evidente pelo valor das médias na pré e pós-intervenção que os juízes avaliaram uma melhora significativa dos participantes em todos os itens analisados. Os itens contato visual (média de 2,26 na pré e 3,55 na pós), sorrir quando apropriado (média de 2,26 na pré e 3,79 na pós-intervenção) e postura adequada durante a apresentação (média de 2,21 na pré e 3,79 na pós-intervenção) foram os três itens de maior impacto, sendo valor de p<0,001 (estatisticamente significante) em todos os itens.

A Tabela 2 apresenta a comparação dos componentes quantitativos. Desses, os itens com maior média de melhoria, segundo os juízes, em suas avaliações pré e pós-intervenção foram a abordagem (média de 7,35 na pré para 9,28 na pós), organização da apresentação (média de 7,73 na pré para 9,44 na pós), linguagem utilizada (média de 7,58 na pré e 9,31 na pós) e forma de apresentar (média de 7,11 na pré e 9,07 na pós-intervenção), sendo valores estatisticamente significantes (p<0,001) na totalidade dos itens.

**Tabela 2:** Comparação dos componentes quantitativos em função do momento de avaliação de avaliação dos avaliadores/juízes.

Componentes quantitativos	Pré					Pós					Valor-p
	Média	DP	P25	Mediana	P75	Média	DP	P25	Mediana	P75	
Planejamento	8,11	1,09	7,33	8,42	9,00	9,60	0,59	9,50	9,67	10,00	**<0,001** *
Objetivo	7,98	1,02	7,33	8,17	8,75	9,53	0,46	9,33	9,67	9,83	<0,001*
Conteúdo	8,00	1,02	7,46	8,08	8,83	9,47	0,56	9,29	9,67	9,83	<0,001*
Abordagem	7,35	1,07	6,63	7,17	7,88	9,28	0,53	8,96	9,33	9,67	<0,001*
Organização	7,73	1,04	6,96	7,83	8,50	9,44	0,59	9,13	9,54	10,00	<0,001*
Recurso Visual	8,01	1,04	7,33	8,08	8,88	9,62	0,57	9,50	9,83	10,00	<0,001*
Forma de Apresentar	7,11	1,03	6,33	7,17	7,67	9,07	0,53	8,73	9,00	9,54	<0,001*
Linguagem	7,58	0,89	7,00	7,67	8,17	9,31	0,41	8,83	9,33	9,67	<0,001*
Geral	7,71	0,98	7,00	7,83	8,17	9,23	0,48	8,96	9,33	9,58	<0,001*

Teste de Wilcoxon

* p<0,05

**Legenda:** DP=desvio padrão; P25=percentil 25; P75=percentil 75

### Autoavaliação

As queixas mais frequentes dos participantes do estudo em relação a falar em público foram: nervosismo (76%), insegurança (74%), ansiedade (68%), não conseguir transmitir/expressar o que quer falar (63%), medo de esquecer ou errar (60%). Na sequência vieram: timidez (53%), saber qual melhor postura (53%), vergonha de falar em público (50%), falar com clareza (50%), projetar corretamente a voz (42%), e outros (8%).

Os resultados apresentados na Tabela 3 demostraram um aumento significativo da pontuação no escore, comparando o pré e o pós-treinamento da subescala SSPS-P e uma redução significativa nos escores pré e pós da subescala SSPS-N, sendo p<0,001 para ambas. Isso ressalta que, na autoavaliação, mais do que o aumento da média das autoafirmativas positivas (média de 15,9 na pré-intervenção e 20,9 na pós-intervenção) foi a diminuição da média das autoafirmações negativas que mais chamou a atenção (média de 10,6 na pré-intervenção para 6,3 na pós-intervenção). Houve também um aumento na porcentagem das autoafirmações positivas que passou de 44% para 68%, bem como diminuição das negativas de 56% para 32% na pós-intervenção.

**Tabela 3:** Comparação das autoafirmações positivas e negativas em função do momento de autoavaliação dos estudantes

Autoafirmações	**Pré**			**Pós**			**P**
	Média	Mediana	DP	Média	Mediana	DP	
SSPS-P	15,9	16,5	4,9	20,9	22	2,9	**<0,001** *
SSPS-N	10,6	10,5	6,7	6,3	5	4,7	<0,001*

Teste de Wilcoxon

* *p*<0,05

**Legenda:** DP= desvio padrão

A Tabela 4 mostra os resultados obtidos na pré e na pós-intervenção na autoavaliação de caráter quantitativo. Esses valores demonstram que os valores atribuídos na pós-intervenção foram maiores que na pré-intervenção, obtendo diferenças estatisticamente significativas em todos os itens (valores de p<0,001), mas com médias melhores expressivamente nos itens organização da apresentação (média de 6,19 na pré para 8,06 na pós), linguagem utilizada (média de 6,03 na pré e 8,11 na pós), recursos visuais utilizados (média de 6,71 na pré e 8,53 na pós-intervenção), controle emocional (média de 5,17 na pré para 7,77 na pós) e na avaliação geral (média de 6,27 na pré para 8,30 na pós-intervenção).

**Tabela 4:** **Comparação dos componentes quantitativos em função do** momento de autoavaliação dos estudantes

Componentes quantitativos	Pré					Pós					Valor-p
	Média	DP	P25	Mediana	P75	Média	DP	P25	Mediana	P75	
Planejamento	7,02	1,98	6,00	7,00	8,25	8,53	1,04	8,00	8,00	9,62	**99**
Conteúdo	7,35	1,63	6,75	8,00	8,12	8,44	0,80	8,00	8,00	9,00	<0,001*
Abordagem	6,13	2,14	5,00	6,00	8,00	7,66	1,14	7,00	8,00	9,00	<0,001*
Organização	6,19	1,75	5,00	6,00	7,00	8,06	1,16	7,00	8,00	9,00	<0,001*
Recurso Visual	6,71	2,24	5,00	7,00	8,00	8,53	1,15	8,00	9,00	9,25	<0,001*
Forma de Apresentar	5,25	2,01	4,00	6,00	7,00	7,60	1,20	7,00	7,00	9,00	<0,001*
Controle Emocional	5,17	2,64	3,00	5,00	8,00	7,77	1,41	7,00	8,00	9,00	**<0,001** *
Linguagem	6,03	1,76	5,00	6,00	7,00	8,11	1,09	7,00	8,00	9,00	<0,001*
Geral	6,27	1,73	5,75	7,00	7,25	8,30	0,90	8,00	8,00	9,00	<0,001*

Teste de Wilcoxon

* p<0,05

Legenda: DP=desvio padrão; P25=percentil 25; P75=percentil 75

## DISCUSSÃO

O programa de treinamento proposto foi efetivo para melhorar as habilidades de comunicação em estudantes universitários. Houve melhora em todas as variáveis mensuradas relacionadas aos recursos verbais e não verbais após o treinamento tanto na avaliação dos juízes quanto na autopercepção dos estudantes. Importante notar que, dos aspectos qualitativos analisados pelos juízes, a melhoria mais ressaltada foi a de contato visual, sorrir quando apropriado e postura adequada durante a apresentação. Quanto aos aspectos quantitativos, foram à abordagem, organização da apresentação, a linguagem utilizada e a forma de apresentar dos participantes.

Embora a melhoria mais acentuada percebida pelos estudantes em suas autoavaliações tenha coincidido, em termos qualitativos, com a avaliação dos juízes nos itens organização da apresentação e linguagem, grande parte desses estudantes teve uma percepção maior na própria melhora do controle emocional e em sua avaliação geral (que inclui a clareza da mensagem, se alcançou os objetivos, se foi interessante, agradável/ informativo ou instigante). Pesquisas anteriores também afirmam que os aspectos anteriormente citados tanto pelos juízes quanto pelos participantes do estudo aqui relatados são complementares, ou seja, as emoções habituais influenciam na expressão facial, na voz e na postura corporal^(9,10)^.

O presente estudo trouxe técnicas e ferramentas de diferentes áreas^(13,14,18,19,23-25)^ integradas em um material didático previamente elaborado, que serviu tanto para a elaboração do treinamento quanto como material de apoio para instruir o uso dos recursos linguísticos nas apresentações orais, em alunos de ensino superior. Especificamente sobre a postura corporal e a expressão facial, no estudo aqui apresentado esses foram dois itens avaliados tanto pelos juízes quanto pelos participantes como itens com melhora acentuada, dado os valores de médias alcançados, expressados pela aumento no contato visual, no uso de sorriso em momentos adequados da apresentação e a postura corporal mantida durante a mesma, quando comparados os momentos de avaliação antes e após o treinamento aqui descrito, o que confirma achados em estudos e trabalhos similares das áreas relacionadas a treinamentos de comunicação^(9,13,14,19)^.

Ao se trabalhar com a comunicação humana, a emoção é um dos fatores importantes para transmitir a intenção de forma convincente. Dessa forma, é uma parte importante de uma comunicação humana natural e espontânea. A literatura^(11,19,23)^ demonstra que, durante a comunicação, a emoção pode ser expressa tanto de forma consciente como inconsciente, por meio de aspectos como o próprio enunciado linguístico^(6)^, características paralinguísticas ou características vocais^(20)^. Esse dado se confirma no estudo aqui apresentado mais especificamente na autoavaliação dos participantes, os quais citam aumento em seu controle emocional, quando comparadas as medias antes e após o treinamento.

Essa autoavaliação positiva do aspecto emocional dos participantes é importante e significativa, dado que as queixas mais frequentes dos participantes do estudo antes de iniciar a intervenção e que pautaram a decisão para se inscreverem e participarem do treinamento foram exatamente o nervosismo, insegurança, ansiedade e não conseguir transmitir/expressar o que quer falar. Conforme estudo anterior^(8)^, essas queixas podem ser decorrentes de falta de prática ao falar, domínio insuficiente do tópico e autoimagem negativa, sendo a falta de experiência o fator mais importante^(1,23)^, o que fez com que o treinamento aqui proposto alcançasse resultados positivos.

Ainda sobre a autoimagem, na autoavaliação realizada pelos participantes e evidenciada por um dos instrumentos^(4)^, as autoafirmações positivas tiveram um acréscimo significativo. As autoafirmações negativas em relação às suas apresentações tiveram um decréscimo também significativo, reforçando os resultados de estudos^(7,8,22)^. Aponta-se nesses estudos que a autoimagem negativa impacta as apresentações orais e que, quando o indivíduo se sente preparado, essa autoimagem tende a melhorar.

Dada essa consideração em relação a autoimagem de um bom comunicador, optou-se por seguir os pressupostos da Programação Neurolinguística - PNL^(13,14,19)^ como uma das ferramentas para elaborar o material didático e o treinamento, além de propor exercitar também a mudança de estado mental^(18,19,26)^, pelo trabalho direcionado às habilidades sociais, trazendo maior segurança e encorajamento na hora da apresentação.

Para que a mudança de estado mental fosse possível^(19,26)^, no treinamento proposto, os participantes tiveram uma parte teórica sobre como uma apresentação deve ser elaborada para diferentes tipos de público e a importância da abrangência de todos. Esse conceito foi posteriormente denominado na avaliação, assim como em estudos similares^(3,12,27)^, como “fazer contato de olhos com todos os presentes”, “capacidade de manter o interesse da audiência” e também foi considerado dentro do critério “geral”. Tais aspectos tiveram similar avaliação quanto à melhora na pós-intervenção tanto pelos juízes quanto pelos próprios participantes, sendo itens com as mudanças mais significativas, como os resultados obtidos nas pesquisas com populações similares mostram^(3,12,27)^.

No treinamento, as habilidades comunicativas foram trabalhadas por meio de atividades práticas, nas quais os participantes realizaram exercícios vocais e corporais, dadas as relações entre voz e competência comunicativa demonstradas em trabalhos da área^(17,20,23)^. Essas atividades simularam situações de exposição oral com treinamento de projeção vocal, pausas, postura, gestos, e outras habilidades linguísticas e comunicativas, como sugerido na literatura^(3,12,16,20,23,24,27-29)^.

Acredita-se que a melhora nos aspectos como articulação, *loudness*, entonação, pausas e da média de tempo de apresentação alcançados no treinamento aqui proposto deve-se à utilização de técnicas vocais específicas que tem como objetivos, segundo trabalhos da área de voz^(17,20)^, melhorar o padrão articulatório, favorecer a projeção vocal, suavizar a emissão e, ainda^(24,28,29)^, melhorar a coordenação pneumofonoarticulatória e promover maior equilíbrio da produção vocal.

Essas habilidades (articulação, *loudness*, contato visual, sorrir quando apropriado, postura adequada, manter a audiência, entonação e pausas) integram a expressividade^(30)^, sendo que o trabalho conjunto com todas essas variáveis possibilita uma comunicação adequada ao contexto e ao discurso. Além disso, possibilita aprimorar a autopercepção sobre si mesmo e sobre a comunicação do falante, o que é corroborado em estudos não somente com populações similares como estudantes universitários^(3,12,27)^, mas com outras populações, inclusive envolvendo outros profissionais^(1,2,7,20)^.

A melhora no vocabulário, na gramática e no uso de pronomes pelos participantes do estudo pode ter ocorrido pelo conteúdo sobre a linguagem abordado no primeiro e último capítulos do material teórico-prático preparado para o treinamento. O material orientava sobre a forma de falar, escolha de palavras, regência, para que os estudantes atentassem a esses aspectos na hora de uma apresentação oral.

Abordar a relação entre todos os aspectos envolvidos na comunicação, como a linguagem, a voz, a competência comunicativa em si, os gêneros linguísticos e não-linguísticos utilizados no processo de se comunicar, assim como enfocar as próprias habilidades sociais^(2,6,13,17,18,20)^ como comportamentos que se podem observar e mensurar, permite uma visão mais global do falante sobre seu desempenho. Retira o foco apenas de questões emocionais ligadas inicialmente pelos participantes como o medo e a ansiedade por falar em público^(1,8,11,16,23,30)^.

A autoavaliação inserida no estudo trouxe resultados importantes, com várias implicações positivas para o indivíduo, como apontado em estudos similares^(1,3,7,22)^, pois facilita a autorregulação, fornece uma sensação de auto continuidade, acelera o processamento de informações relevantes, auxilia no estabelecimento de metas, influencia a percepção social, e contribui para a projeção de uma autoimagem consistente e desejável para os outros^(3)^.

A melhoria indicada pelos participantes dessa pesquisa nos aspectos de “Planejamento”, “Objetivos”, “Conteúdo”, “Abordagem”, “Organização” e “Recursos visuais” de suas autoavaliações, seja devido ao que foi abordado no treinamento sobre como estruturar uma pesquisa e uma apresentação (utilizando conceitos básicos de metodologia de pesquisa e design), seja na ênfase dada à uma preparação teórica e prática da apresentação, confirmou estudos que mostram que essa prévia preparação sobre o tema - como domínio do assunto, a coerência, o embasamento científico e a forma de conduzir a apresentação - pode diminuir até 75% o medo de falar em público.

O conhecimento do conteúdo afeta diretamente o estilo como ele é conduzido pelo apresentador, podendo demonstrar maior ou menor grau de segurança e manejo crítico do conteúdo, de forma a envolver a audiência, conforme afirmam trabalhos anteriores^(25,30)^.

Outro aspecto a ser discutido é a Escala para Autoavaliação ao Falar em Público (SSPS), pois uma das limitações desse estudo foi o fato de não serem encontrados questionários validados que contemplassem os itens necessários para avaliar a performance dos participantes de acordo com a necessidade do estudo, portanto a escala SSPS foi traduzida e adaptada às necessidades da pesquisa.

Quanto aos resultados de aplicação da escala, analisando os seus escores, evidencia-se que, ao aumentar a pontuação da subescala SSPS-P (auto afirmativas positivas) e, ao diminuir a da SSPS-N (auto afirmativas negativas), os participantes se tornaram mais autoconfiantes e capazes de enfrentar a situação de exposição em público e também demostraram estar menos ansiosos, mais autoconfiantes e preparados ao passarem pelo treinamento de comunicação.

Estudos^(4,19)^ que utilizaram a mesma escala ressaltam que as autoafirmações negativas podem estar mais intimamente associadas à ansiedade de falar em público do que as positivas. Isso pode apontar que os participantes, ao pontuarem menos as autoafirmações negativas, tornam-se menos propensos a reafirmar ou aumentar sua ansiedade ao falar em público e até mesmo desenvolver uma fobia social ou transtornos de ansiedade relacionados à isso. Estudos na área^(8,12-14,19,22,23,26)^ mostram que treinamentos de exposição em público e de comunicação, assim como técnicas cognitivas-comportamentais e treinamento em habilidades sociais são eficazes para o tratamento da fobia social relacionada ao medo de falar em público.

Em um estudo^(27)^ que tinha como objetivo analisar uma intervenção fonoaudiológica junto a estudantes universitários em situações de apresentações de seminários, os resultados encontrados mostraram efeitos positivos da intervenção nos aspectos orais, além de melhora na percepção de segurança relatadas pelos participantes quando conseguiam atingir melhor suas plateias. As fonoaudiólogas avaliadoras do estudo citado perceberam avanços quanto aos aspectos orais, corporais e interacionais. Assim, os resultados gerais da pesquisa aqui apresentada corroboram os achados desse estudo^(27)^ e abrem uma reflexão sobre o porquê desse medo ainda ser um dos mais prevalentes entre os universitários. Não seria papel das universidades prepararem os alunos para tal atividade?

A cada ano, milhares de estudantes são admitidos nas universidades em busca de uma profissão e, no decorrer dos anos de formação, encontram tarefas que demandam crescimento intelectual e aprimoramento de habilidades, tais como a de falar em público. Se uma Instituição de Ensino Superior entende que é preciso preparar seus alunos para se comunicarem com eficiência em seus futuros ambientes de atuação profissional^(1-3)^, cabe refletir sobre como tem procedido quanto a essa formação.

Programas de intervenção sobre oratória e melhora do desempenho da comunicação oral não deveriam integrar o currículo de formação profissional? A formação de profissionais para o mercado de trabalho deveria privilegiar o ensino de competências e habilidades para além dos conteúdos técnicos de determinada profissão. É necessário que as universidades proporcionem condições para que o aluno possa conhecer, além do conteúdo da profissão, as exigências comportamentais da função escolhida^(18)^.

O presente estudo teve um limitador por ter sido realizado com estudante universitários de uma única instituição de ensino (embora de três cursos de graduação diferentes), mas como seus participantes melhoraram seu desempenho em todos os aspectos analisados após passarem pela intervenção, demonstrou-se a aplicabilidade do treinamento proposto enquanto ferramenta auxiliar no planejamento e aprimoramento de treinamentos de comunicação para tal população. Portanto, fica aqui a contribuição dessa pesquisa para que sejam propostos por fonoaudiólogos nas universidades programas de treinamento e consultoria fonoaudiológica na área, sendo esse um campo promissor de atuação profissional.

Sugerem-se estudos similares que utilizem o treinamento aqui proposto e sejam desenvolvidos em outras instituições de Ensino Superior, para que esse estudo contribua para o ensino de competências e habilidades além dos conteúdos técnicos dentro das universidades. Acredita-se também que o material elaborado possa integrar conteúdo de disciplinas em várias universidades e ser um suporte para os universitários na área de comunicação.

## CONCLUSÃO

O programa de treinamento proposto foi efetivo para melhorar as habilidades de comunicação de estudantes universitários em apresentações orais. Houve melhora - tanto da perspectiva dos juízes avaliadores quanto da dos próprios participantes - nas habilidades relacionadas aos recursos verbais e não verbais utilizados, nos aspectos qualitativos como contato visual, sorrir quando apropriado e postura adequada durante a apresentação, bem como nos aspectos quantitativos como organização da apresentação, a linguagem utilizada e o uso de recursos visuais.

Na autoavaliação, os estudantes participantes do estudo também relataram melhoria do controle emocional durante as apresentações orais e em suas avaliações gerais após o treinamento. que inclui a clareza da mensagem, se esta alcançou os objetivos, se foi interessante, agradável/ informativo ou instigante.

## **Anexo 1.** Ficha de cadastro inicial

**Table:**

**Nome completo:**
**Data de Nascimento: Nº USP:**
**Endereço:**
**Bairro:**
**Cidade: UF:**
**Telefone: ( ) Celular: ( )**
**E-mail:**
**Curso: Ano de Graduação:**
**Pode vir realizar a pesquisa no horário noturno (19-21h) com frequência de uma vez por semana em oito encontros, na xxxxxxxxxxxxxx, em dia a combinar?**
**( ) Sim ( ) Não**
**Qual dia da semana de sua preferência para a realização da pesquisa?**
**( ) Segunda ( ) Terça ( ) Quarta ( ) Quinta ( ) Sexta**
**Principais dificuldades nas apresentações:**
**( ) Nervosismo ( ) Ansiedade ( ) Insegurança ( ) Timidez ( ) Não conseguir transmitir/expressar o que quero falar ( ) Medo de esquecer ou errar ( ) Vergonha de falar em público ( ) Falar com clareza ( ) Projetar corretamente a minha voz ( ) Em saber qual a melhor postura ( ) Outros**
**Se assinalar outros, descrever aqui:**

**Esta dificuldade é ou foi notada por quem? (pode marcar mais de uma opção):**
**( ) por você mesmo ( ) professor da faculdade ( ) outro professor ( ) colegas de classe ( ) amigos de outros locais ( ) família ( )outros/quem:**

## **Anexo 2**. Formulário de Avaliação dos Juízes - FAO

Apresentação Oral

Nome da Avaliadora:

Nome do Avaliado:

Data:

1. Formulário de avaliação da apresentação oral

Parte I- Aspectos a serem analisados.

1.1.1. Critérios Linguísticos - Aspectos Formais e informais:

Faz uso correto do (a):

**Table:**

**Frequência**	**Sempre**	**Frequentemente**	**Às vezes**	**Raramente**	**Nunca**
(a) Vocabulário					
(b) Gramática					
(c) Pronúncia					
(d) Entonação					
(e) pausas					
(f) Média de tempo					

1.1.2. Aspectos complementares a comunicação (não verbais), faz uso dos aspectos abaixo:

**Table:**

**Frequência**	**Sempre**	**Frequentemente**	**Ás vezes**	**Raramente**	**Nunca**
(a) Articulação					
(b) *Loudness*					
(c) Fazer contato visual com todos os presentes					
(d) Sorrir quando apropriado					
(e) Postura adequada					
(f) Capacidade de manter o interesse da audiência					
(g) Controle Emocional					

Parte II

De 0 a 10 atribuía uma nota para cada aspecto abaixo. Sendo 0 Despreparado e 10 Excelente.

**Table:**

**Aspectos**	**Pontos a considerar**	**Nota**
Planejamento	Evidência de preparação cuidadosa.	
Objetivos	Clareza: adequados à audiência ou ao assunto.	
Conteúdo	Alcance, relevância, adequação, conhecimento, pesquisa e domínio do assunto.	
Abordagem	Mensagem, embasamento e reiteração, variedade e humor.	
Organização	Coerência, clareza e condução.	
Recursos visuais	Propriedade, clareza e condução.	
Forma de apresentar	Ritmo, entusiasmo, contato visual, fala audível, entonação, autoconfiança e linguagem corporal.	
Linguagem	Clareza, acuidade, fluência, adequação, pronúncia e sinalização.	
Geral	Clareza da mensagem. Alcançou os objetivos? É interessante? Agradável/ informativo? Instigante?	

## **Anexo 3**. Formulário de Autoavaliação

Apresentação Oral

Nome: Data:

Anexo 1. Escala para Auto-Avaliação ao Falar em Público (SSPS) - Versão para o português

**Table:**

Escala para Auto-Avalíação ao Falar em Público Tradução e adaptação para o português: Crippa JAS, Osório F, Graett FG,Zuardi AW, Pinho M, Busano GF, Chaves M, Loureiro SR (2004) Por favor, imagine as coisas que você costuma pensar sobre si mesmo, quando se encontra em alguma situação em que tenha que falar em público. Tendo em mente essas situações, até que ponto você concorda com as afirmações a seguir? Por favor, dê uma nota de O (se você discorda totalmente) a 5 (se você concorda inteiramente com a afirmação)						
1. 0 que tenho a perder? Vale a pena tentar	0	1	2	3	4	5
2. Sou um fracasso	0	1	2	3	4	5
3. Esta é uma situação difícil, mas posso dar conta dela	0	1	2	3	4	5
4. Um fracasso nesta situação seria mais uma prova de minha incompetência	0	1	2	3	4	5
5. Mesmo que não dê certo, não é o fim do mundo	0	1	2	3	4	5
6. Posso dar conta de tudo	0	1	2	3	4	5
7. Qualquer coisa que eu disser vai parecer bobagem	0	1	2	3	4	5
8. Acho que vou me dar mal de qualquer jeito	0	1	2	3	4	5
9. Em vez de me preocupar, poderia me concentrar no que quero dizer	0	1	2	3	4	5
10. Eu me sinto desajeitado e tolo, certamente eles vão notar	0	1	2	3	4	5

*Copyright Stetan G. Hotmann.*

2. Avaliação geral

De 0 a 10 atribuía uma nota para cada aspecto abaixo. Sendo 0 Despreparado e 10 Excelente.

**Table:**

Aspectos	Pontos a considerar	Nota
Planejamento	Evidência de preparação cuidadosa.	
Objetivos	Clareza: adequados à audiência ou ao assunto.	
Conteúdo	Alcance, relevância, adequação, conhecimento, pesquisa e domínio do assunto.	
Abordagem	Mensagem, embasamento e reiteração, variedade e humor.	
Organização	Coerência, clareza e condução.	
Recursos visuais	Propriedade, clareza e condução.	
Forma de apresentar	Ritmo, entusiasmo, contato visual, fala audível, entonação, autoconfiança e linguagem corporal.	
Linguagem	Clareza, acuidade, fluência, adequação, pronúncia e sinalização.	
Geral	Clareza da mensagem. Alcançou os objetivos? É interessante? Agradável/ informativo? Instigante?
